# Supplementary material for: The interactome of intact mitochondria by cross-linking mass spectrometry provides evidence for coexisting respiratory supercomplexes
Source: Mol Cell Proteomics. 2017 Dec 8;17(2):216–32. doi: 10.1074/mcp.RA117.000470 (PMC5795388; doi:10.1074/mcp.RA117.000470)
Supplement: Supplemental Data [file supp_RA117.000470_133922_0_supp_23978_fzffwf.zip › spectra_annotation/mito_DR_spectra_annotation/64-1-7-1-6-1.pdf]

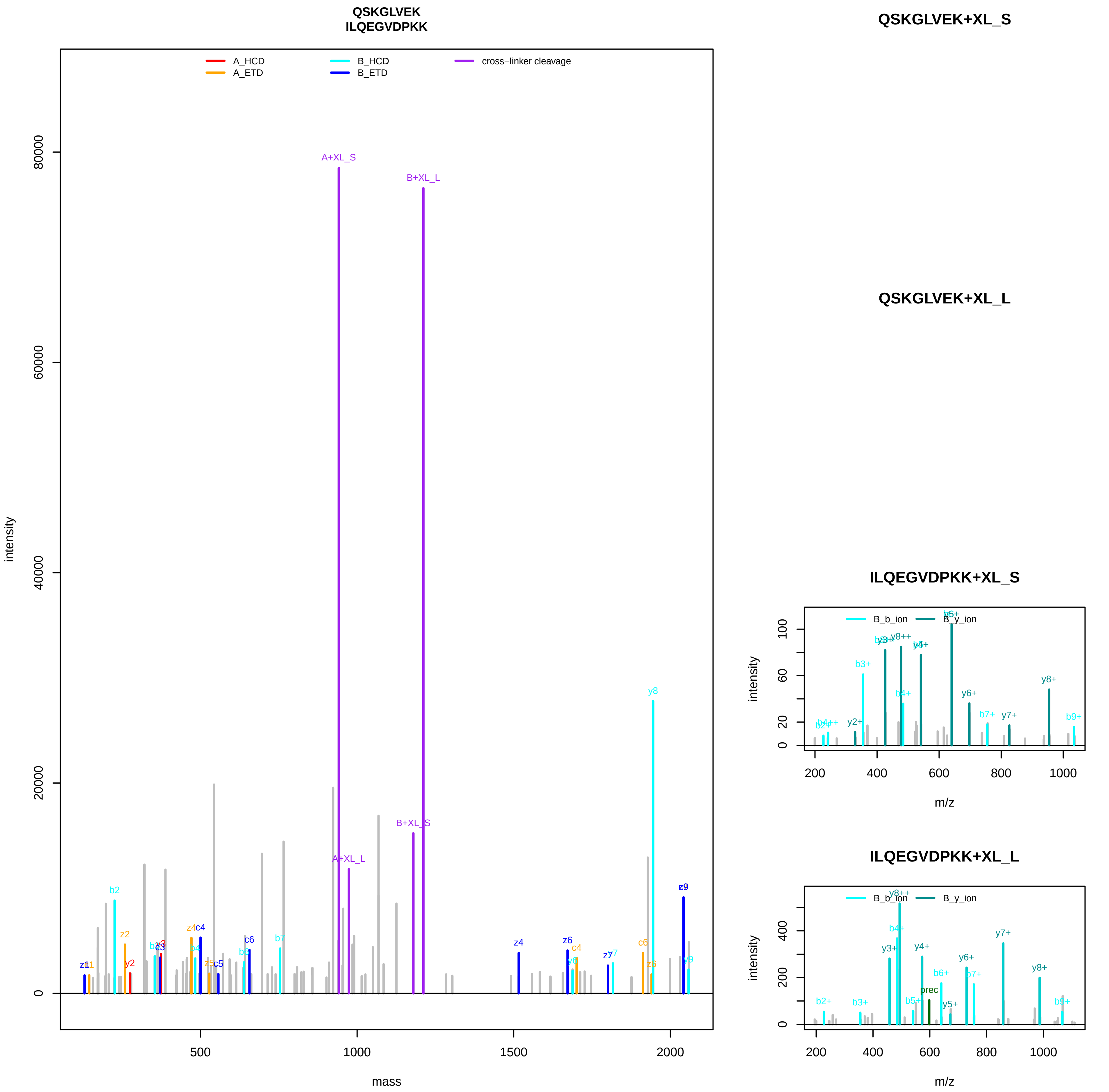

QSKGLVEK
ILQEGVDPKK
A_HCD
A_ETD
B_HCD
B_ETD
cross−linker cleavage
A+XL_S
B+XL_L
B+XL_S
A+XL_L
y8
c9
b2
z2
y2
y3
c3
z4
c4
b4
z5
c5
c6
b7
z4
z6
c4
z7
c6
z6
y9
intensity
mass
0
20000
40000
60000
80000
500
1000
1500
2000
QSKGLVEK+XL_S
QSKGLVEK+XL_L
ILQEGVDPKK+XL_S
B_b_ion
B_y_ion
y5+
y3+
y8++
y4+
b3+
b4+
y6+
y8+
b7+
y7+
b9+
b2+
b4++
y2+
intensity
m/z
0
20
60
100
200
400
600
800
1000
ILQEGVDPKK+XL_L
B_b_ion
B_y_ion
y8++
b4+
y3+
y4+
y7+
y6+
b6+
b7+
y8+
prec
b2+
b3+
b5+
y5+
b9+
intensity
m/z
0
200
400
200
400
600
800
1000
